# Pirfenidone vs. nintedanib in patients with idiopathic pulmonary fibrosis: a retrospective cohort study

**DOI:** 10.1186/s12931-021-01857-y

**Published:** 2021-10-19

**Authors:** Pavo Marijic, Larissa Schwarzkopf, Lars Schwettmann, Thomas Ruhnke, Franziska Trudzinski, Michael Kreuter

**Affiliations:** 1 Institute of Health Economics and Health Care Management, Helmholtz Zentrum München - German Research Center for Environmental Health (GmbH), Neuherberg, Germany; 2 Pettenkoffer School of Public Health, Munich, Germany; 3 Institute for Medical Information Processing, Biometry and Epidemiology - IBE, LMU Munich, Munich, Germany; 4 Comprehensive Pneumology Center Munich (CPC-M), Member of the German Center for Lung Research (DZL), Munich, Germany; 5 IFT-Institut Fuer Therapieforschung, Munich, Germany; 6 Department of Economics, Martin Luther University Halle-Wittenberg, Halle, Germany; 7 AOK Research Institute - WIdO, Berlin, Germany; 8 Center for Interstitial and Rare Lung Diseases, Pneumology and Respiratory Critical Care Medicine, Thoraxklinik, University of Heidelberg, Member of the German Center for Lung Research (DZL), Heidelberg, Germany

**Keywords:** Idiopathic pulmonary fibrosis, Mortality, Hospitalization, Health care costs, Administrative data, Drugs, Statutory health insurance

## Abstract

**Background:**

Two antifibrotic drugs, pirfenidone and nintedanib, are licensed for the treatment of patients with idiopathic pulmonary fibrosis (IPF). However, there is neither evidence from prospective data nor a guideline recommendation, which drug should be preferred over the other. This study aimed to compare pirfenidone and nintedanib-treated patients regarding all-cause mortality, all-cause and respiratory-related hospitalizations, and overall as well as respiratory-related health care costs borne by the Statutory Health Insurance (SHI).

**Methods:**

A retrospective cohort study with SHI data was performed, including IPF patients treated either with pirfenidone or nintedanib. Stabilized inverse probability of treatment weighting (IPTW) based on propensity scores was applied to adjust for observed covariates. Weighted Cox models were estimated to analyze mortality and hospitalization. Weighted cost differences with bootstrapped 95% confidence intervals (CI) were applied for cost analysis.

**Results:**

We compared 840 patients treated with pirfenidone and 713 patients treated with nintedanib. Both groups were similar regarding two-year all-cause mortality (HR: 0.90 95% CI: 0.76; 1.07), one-year all cause (HR: 1.09, 95% CI: 0.95; 1.25) and respiratory-related hospitalization (HR: 0.89, 95% CI: 0.72; 1.08). No significant differences were observed regarding total (€− 807, 95% CI: €− 2977; €1220) and respiratory-related (€− 1282, 95% CI: €− 3423; €534) costs.

**Conclusion:**

Our analyses suggest that the patient-related outcomes mortality, hospitalization, and costs do not differ between the two currently available antifibrotic drugs pirfenidone and nintedanib. Hence, the decision on treatment with pirfenidone versus treatment with nintedanib ought to be made case-by-case taking clinical characteristics, comorbidities, comedications, individual risk of side effects, and patients’ preferences into account.

**Supplementary Information:**

The online version contains supplementary material available at 10.1186/s12931-021-01857-y.

## Background

Idiopathic pulmonary fibrosis (IPF) is a chronic, progressive, fibrosing interstitial lung disease (ILD) of unknown etiology [1]. The prognosis of survival is poor, with a reported median survival time of 3.8 years [2]. In recent years, two antifibrotic drugs, viz. pirfenidone and nintedanib, have been licensed for treatment of IPF patients. In Germany, pirfenidone was approved in 2012 and nintedanib in 2015. Both drugs have been shown to slow IPF progression [3, 4], to increase survival [5–7] and to reduce respiratory-related hospitalizations [8]. A recently published systematic review and meta-analysis of RCTs and cohort studies reported that antifibrotic treatment appears to reduce the risk of mortality and acute exacerbations [9]. So far, however, there is no guideline recommendation which drug should be preferred over the other [10], as no randomized controlled trials (RCT) comparing the two drugs have been conducted. Comparative observational studies, especially in real world settings are also rare and show inconclusive results on survival differences between the drugs [11–13]. A claims data-based study among Medicare enrollees suggested that pirfenidone treatment is associated with fewer all-cause and fewer respiratory-related hospitalizations and thus lower inpatient costs compared to nintedanib treatment [15]. Although the study included a large sample, it had a short observation period and did not compare mortality differences between the drugs. A recently published study with data from the French National Health System [13] also reported an association between pirfenidone treatment and lower respiratory-related hospitalizations. However, all-cause hospitalizations were not investigated.

Hence, given the inconclusive and sparse evidence, there is need to further investigate potential differences between both drugs regarding their effectiveness and related costs to guide clinicians with their treatment decisions. To broaden the pre-existing body of evidence, this study aimed to compare pirfenidone and nintedanib-treated patients regarding all-cause mortality, all-cause as well as respiratory-related hospitalization, and overall as well as respiratory-related health care costs.

## Methods

### Data set and sample selection

We performed a retrospective cohort study with health insurance claims data of the Allgemeine Ortskrankenkasse (AOK) provided by the AOK Research Institute. AOK provides statutory health insurance for roughly 32 percent of the German population [16]. Membership is open to anyone regardless of factors such as professional affiliation, income, age or comorbidities [17].

The initial data set included all individuals insured with an ICD-10 diagnosis of various ILDs, including IPF [J84.1], other fibrosing ILDs [J84.0, J84.8, J84.9, D48.1], sarcoidosis [D86.0-D86.9], drug-associated ILDs [J70.2-J70.4], pneumoconiosis [J62.0-J62.8, J63.0-J63.8], radiation-associated pneumonitis [J70.1], eosinophilic pneumonia [J82], hypersensitivity pneumonitis [J67.9] and connective tissue-associated ILD [J99.1] from January 1, 2013 to December 31, 2018. Survival information was available until the end of 2019. For our analysis, we selected a subsample with at least one IPF diagnosis [J84.1] combined with at least one prescription of pirfenidone (tradename: “Esbriet”) or nintedanib (tradename: “Ofev”) in the patient-individual observation period. Furthermore, selected patients needed to be at least 40 years old at the date of the therapy initiation.

To identify relevant patients for our analysis we used the ATC-Codes “L04AX05” for pirfenidone and “L01XE31” for nintedanib. For the latter, only “Ofev” was considered via the national product codes (Pharmazentralnummer—PZN), as nintedanib under the tradename “Vargatev” is also licensed for treating distinct forms of non-small-cell lung cancer. Furthermore, the license of nintedanib for systemic sclerosis-associated interstitial lung disease and progressive fibrosing interstitial lung disease is not considered in this study as the approval took place after the study period.

We subsequently excluded patients, who were not continuously insured with the AOK and those with a baseline period (pre-observational) or an outcome period (post-observational) of less than one year. Hence, we dropped cases with a first prescription before January 1, 2014 or after December 31, 2017.

Therapy initiation was set as the date the patient redeemed the first drug prescription with either pirfenidone or nintedanib. To perform an intention-to-treat analysis group assignment was based on the first prescription.

### Outcome variables

We compared pirfenidone and nintedanib-treated patients regarding two-year all-cause mortality, one-year all-cause as well as respiratory-related hospitalization, and one year overall and respiratory-related health care costs. All outcomes were calculated starting at the date of the treatment initiation.

Respiratory-related hospitalizations included all hospital visits with the following ICD-10 codes as primary diagnosis [18]: IPF [J84.1], respiratory infection [A481, B250, J09-J22, J40], pneumothorax [J93], pulmonary embolism [I26], pulmonary hypertension and right heart disease [I50, I270, I272, I278, I279], respiratory insufficiency [J96], and other chronic and acute lung diseases [J40-J47].

All-cause and respiratory-related health care costs in the year after therapy initiation were calculated based on outpatient physician costs, inpatient costs, and pharmaceutical costs, which could be directly obtained from the claims data. Respiratory-related costs in the inpatient and outpatient sector were based on cases with the diagnoses mentioned above. For respiratory-related pharmaceutical costs we filed prescriptions of pirfenidone, nintedanib, glucocorticoids, corticosteroids, immunosuppressants, acetylcysteine, sildenafil, and antihypertensives for pulmonary arterial hypertension. The corresponding ATC-Codes are presented in Additional file 1: Table S1. Outpatient physician costs were available on a quarterly basis only. Therefore, we redistributed costs incurred in the quarter of the treatment initiation proportionally to the time before and after the date of treatment initiation. Accordingly, this was also carried out for the last observation quarter. Inpatient costs could be determined on a daily base, but if a hospital stay exceeded the one-year follow-up time, costs were also distributed proportionally to in hospital days within the observation period. Pharmaceutical costs were calculated based on the day they were retrieved by the patient.

### Covariates and stabilized inverse probability of treatment weighting

Stabilized inverse probability of treatment weighting (IPTW) based on propensity scores [19, 20] was used to adjust for differences in covariates between both groups, as no randomization was performed. The advantage of IPTW in comparison to k:1 matching is that all eligible patients retain in the analysis [21]. Covariates included in the estimation of the propensity scores were selected a priori based on clinical expertise and pre-existing literature. These covariates comprised age, gender, and residential area in four district types (major city, urban, rural, remote rural) [22]. Furthermore, area deprivation according to the well-established “German Index of Multiple Deprivation” from the year 2010 (GIMD 2010) was incorporated, which usually serves as a proxy for socioeconomic background if corresponding individual data is not available [23, 24]. Additionally, the time (in years) between the first IPF diagnosis (left-censored at 2013) and treatment initiation was taken into account. Comorbidities were considered via the Elixhauser Index [25] using the ICD-10 coding algorithm of Quan et al. [26]. To capture IPF-relevant comorbidities more precisely, we modified the Elixhauser Index as described by Schwarzkopf et al. [27]. Accordingly, we separated pulmonary hypertension and lung cancer from the corresponding Elixhauser categories and analyzed them separately. The remaining Elixhauser categories were included in the propensity score model, if they had a prevalence of at least 5% in at least one treatment group. In addition, we included other IPF-specific comorbidities not covered by the Elixhauser Index [27], namely gastro-oesophageal reflux disease [K21], obstructive sleep apnoea syndrome [G47.3], ischemic heart disease [I20-I25], and thromboembolism [I80, I26]. To avoid false-positive comorbidity diagnoses, patients had to have at least two confirmed outpatient physician diagnosis in two separated quarters or one primary inpatient diagnosis in the year before therapy initiation. We accounted for the intake of IPF-related or comorbidity-specific drugs in the six months prior to therapy initiation. IPF-related drugs included immunosuppressants, acetylcysteine, glucocorticoids, and corticosteroids. Comorbidity-specific drugs followed the prescription patterns described by Schwarzkopf et al. [27] with corresponding ATC-Codes presented in Additional file 1: Table S1. Furthermore, we considered the use of health care services before therapy initiation by reflecting IPF-specific hospitalization (IPF as primary diagnosis), respiratory-related hospitalization, and all-cause hospitalization in the three months before therapy initiation by corresponding binary variables. Also included were the number of contacts to outpatient pulmonologists and the number of overall contacts to outpatient physicians in the year before therapy initiation.

Standardized Mean Differences (SMD) were used to assess balance of the covariates between the groups after IPTW, with differences less than 0.1 indicating a good balance [28].

### Statistical analysis

Mortality was analyzed by using IPTW-weighted mortality rates per 100 person-years and Kaplan–Meier plots, while hospitalization was investigated with IPTW-weighted hospitalization rates per 100 person-years and cumulative probability curves. Additionally, we calculated weighted Cox Proportional Hazard models with sandwich estimators of the variance for mortality and hospitalization to obtain hazard ratios. In addition, we investigated the three most common reasons for all-cause and respiratory-related hospitalizations. To compare health care costs for the year after treatment initiation, we calculated IPTW-weighted means with bootstrapped 95% confidence intervals (CI). Weighted group differences were also calculated with bootstrapped 95% confidence intervals.

We performed two sensitivity analyses (SA). For SA1, we excluded patients with a first pirfenidone prescription before 2015. Thus, we cover only the period, when both drugs were approved and excluded pirfenidone-treated patients with treatment initiation before nintedanib was available. For SA2, we considered treatment discontinuation, which was defined as a treatment gap of more than 60 days, with day 60 as discontinuation date [15]. Hence, treatment discontinuation became an additional censoring event in the time-to-event analyses. Additionally, we investigated how many of the patients discontinuing the treatment switched medication within the treatment gap range of 60 days. In the cost analysis we excluded costs after the discontinuation date. To account for the discontinued observation time in the cost analysis, we calculated costs per month.

Results were defined as significant if the calculated CIs of the differences did not contain “1” in the Cox models and the “0” in the cost analyses. All analyses were conducted with R-Software version 4.0.3.

## Results

### Population characteristics

**Fig. 1:**
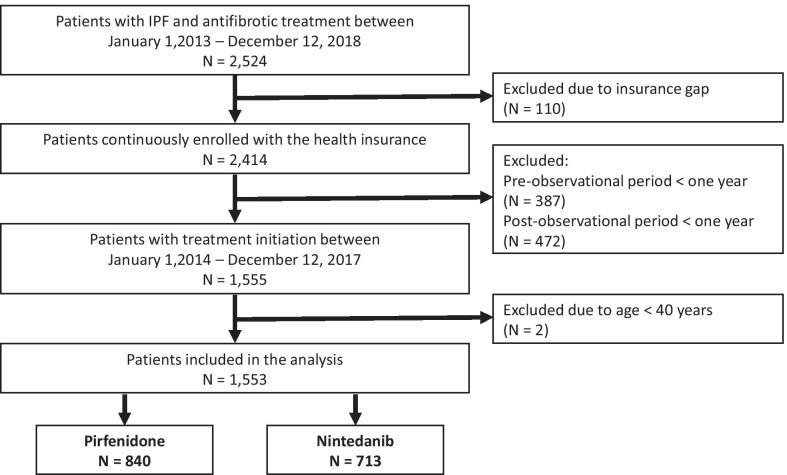
Participant flow of the study population

We identified 2524 patients with an IPF diagnosis who received an antifibrotic therapy with nintedanib or pirfenidone between January 1, 2013 and December 31, 2018. We excluded 110 patients with an insurance gap during the observation period and 859 patients with too short pre or post-observation period. In addition, two patients younger than 40 years were excluded. In total, eligibility criteria were met by 1553 patients, of whom 840 initially received pirfenidone (54.1%) (Fig. 1).

Even before the weighting process, both populations were balanced in most baseline variables (Table 1). Nintedanib-treated patients were slightly older than pirfenidone-treated patients. Pirfenidone-treated patients presented a shorter latency from diagnosis to treatment. Apart from few exceptions, the comorbidity profile was similar in both groups. Pirfenidone-treated patients had however more often cardiac arrhythmias and accordingly a higher probability of being treated with related drugs, while nintedanib-treated patients more often suffered from lung cancer. Furthermore, pirfenidone-treated patients were more often treated with acetylcysteine and anti-clotting drugs. After weighting SMDs for all variables were lower than 0.1 indicating no differences in the covariable structure.

**Table 1:** Patients characteristics

	Unweighted			IPTW-weighted
	Pirfenidone (N = 840)	Nintedanib (N = 713)	SMD	SMD
Age (years), mean (SD)	71.9 (8.6)	73.0 (8.7)	0.121	0.013
Sex (female), n (%)	207 (24.6)	190 (26.6)	0.046	0.005
GIMD 2010 n (%)				
Q1 (least deprived quintile)	202 (24.0)	165 (23.1)	0.075	0.015
Q2	194 (23.1)	170 (23.8)		
Q3	155 (18.5)	138 (19.4)		
Q4	163 (19.4)	129 (18.1)		
Q5 (most deprived quintile)	114 (13.6)	95 (13.3)		
Unknown	12 (1.4)	16 (2.2)		
Residential area n (%)				
Major city	176 (21.0)	154 (21.6)	0.076	0.018
Urban districts	341 (40.6)	268 (37.6)		
Rural districts	165 (19.6)	140 (19.6)		
Remote rural districts	155 (18.5)	149 (20.9)		
Unknown	3 (0.4)	2 (0.3)		
Latency from diagnosis to treatment (years), mean (SD)	0.8 (1.0)	1.2 (1.3)	0.358	0.002
Comorbidities Elixhauser score, mean (SD)	3.4 (2.2)	3.4 (2.1)	0.033	0.002
Comorbidities modified Elixhauser categories, n (%)				
Congestive heart failure	199 (23.7)	157 (22.0)	0.040	< 0.001
Cardiac arrhythmias	207 (24.6)	122 (17.1)	0.186	0.001
Valvular disease	110 (13.1)	90 (12.6)	0.014	0.005
Peripheral vascular disorders	166 (19.8)	140 (19.6)	0.003	0.007
Hypertension, uncomplicated	452 (53.8)	404 (56.7)	0.057	0.006
Hypertension, complicated	107 (12.7)	85 (11.9)	0.025	0.006
Chronic pulmonary disease	352 (41.9)	308 (43.2)	0.026	0.005
Diabetes, uncomplicated	136 (16.2)	91 (12.8)	0.098	0.004
Diabetes, complicated	168 (20.0)	156 (21.9)	0.046	0.006
Hypothyroidism	73 (8.7)	68 (9.5)	0.029	0.009
Renal failure	120 (14.3)	107 (15.0)	0.020	0.020
Liver disease	126 (15.0)	96 (13.5)	0.044	< 0.001
Solid tumor without metastasis	82 (9.8)	61 (8.6)	0.042	0.010
Rheumatoid arthritis/collagen vascular diseases	77 (9.2)	63 (8.8)	0.012	0.011
Obesity	154 (18.3)	150 (21.0)	0.068	0.005
Depression	160 (19.0)	131 (18.4)	0.017	0.006
Comorbidities IPF-specific, n (%)				
Coronary heart disease	318 (37.9)	271 (38.0)	0.003	0.003
Gastro-oesophageal reflux disease	167 (19.9)	123 (17.3)	0.068	0.008
Obstructive sleep apnoea syndrome	86 (10.2)	59 (8.3)	0.068	0.003
Thrombosis	31 (3.7)	28 (3.9)	0.012	0.020
Lung cancer	9 (1.1)	16 (2.2)	0.092	0.011
Pulmonary hypertension	43 (5.1)	34 (4.8)	0.016	0.011
Drug treatments, n (%)				
Immunosuppressants	35 (4.2)	22 (3.1)	0.058	0.002
Acetylcysteine	142 (16.9)	71 (10.0)	0.205	0.001
Glucocorticoids, Corticosteroids	323 (38.5)	274 (38.4)	< 0.001	0.005
Treatment with anti-clotting drugs	250 (29.8)	166 (23.3)	0.147	0.002
Treatment with anti-acid drugs	476 (56.7)	387 (54.3)	0.048	0.004
Treatment with anti-depressants	99 (11.8)	84 (11.8)	< 0.001	0.002
Treatment with anti-diabetic drugs	201 (23.9)	160 (22.4)	0.035	0.001
Treatment with drugs against obstructive airway disease	219 (26.1)	210 (29.5)	0.076	0.004
Treatment of heart insufficiency/cardiac arrhythmia	348 (41.4)	244 (34.2)	0.149	0.001
Treatment of cardiovascular disease	601 (71.5)	483 (67.7)	0.083	0.007
Hospitalizations in three months before treatment, n (%)				
All cause	611 (72.7)	486 (68.2)	0.100	< 0.001
IPF-specific	473 (56.3)	384 (53.9)	0.049	0.005
Respiratory-related	79 (9.4)	64 (9.0)	0.015	0.007
Use of outpatient services in the year before treatment, mean (SD)				
Contacts to physicians overall	19.2 (8.5)	18.6 (7.9)	0.072	0.003
Contact to pulmonologists	2.0 (1.8)	2.1 (1.9)	0.077	0.009

*GIMD 2010* German Index of Multiple Deprivation, year 2010, *Q* Quintile, *SD* Standard deviation, *SMD* Standardized mean difference

### Mortality

**Fig. 2:**
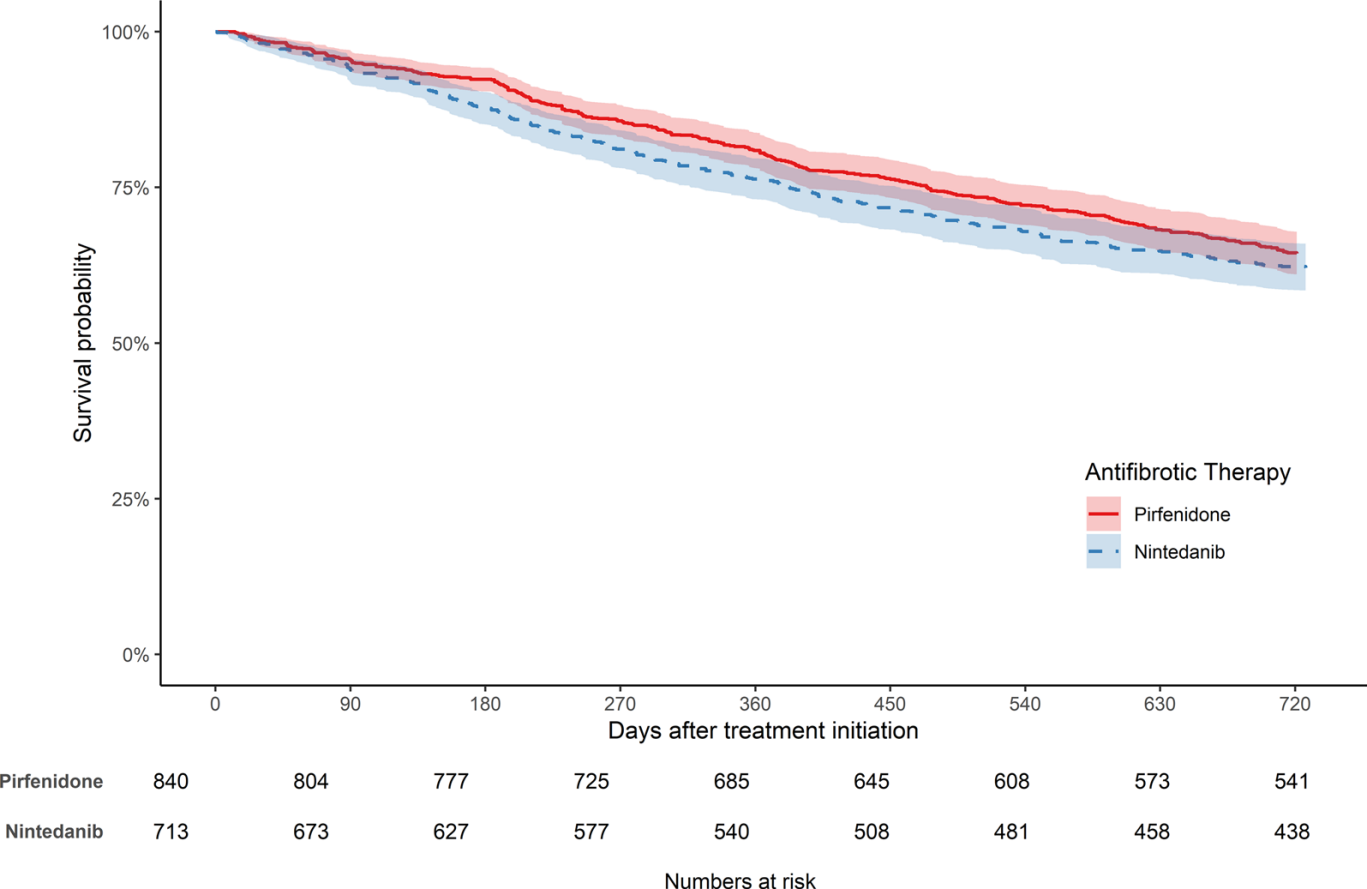
IPTW-weighted Kaplan–Meier plots for two-year all-cause mortality in patients treated with pirfenidone or nintedanib. The colored areas represent 95% confidence intervals

About 36% of pirfenidone-treated and 39% of the nintedanib-treated patients died during the two years after treatment initiation. Weighted mortality rates were 21.9 (95% CI: 19.5; 24.6) per 100 person-years for pirfenidone-treated and 24.4 (95% CI: 21.6; 27.5) for nintedanib-treated patients. Weighted two-year mortality after initiation of antifibrotic treatment was similar in both groups (Fig. 2). Accordingly, the weighted hazard ratio (HR) was not significant (0.90, 95% CI: 0.76; 1.07). The unweighted results are presented in Additional file 2: Tables S2, S3.

### Hospitalization

**Fig. 3:**
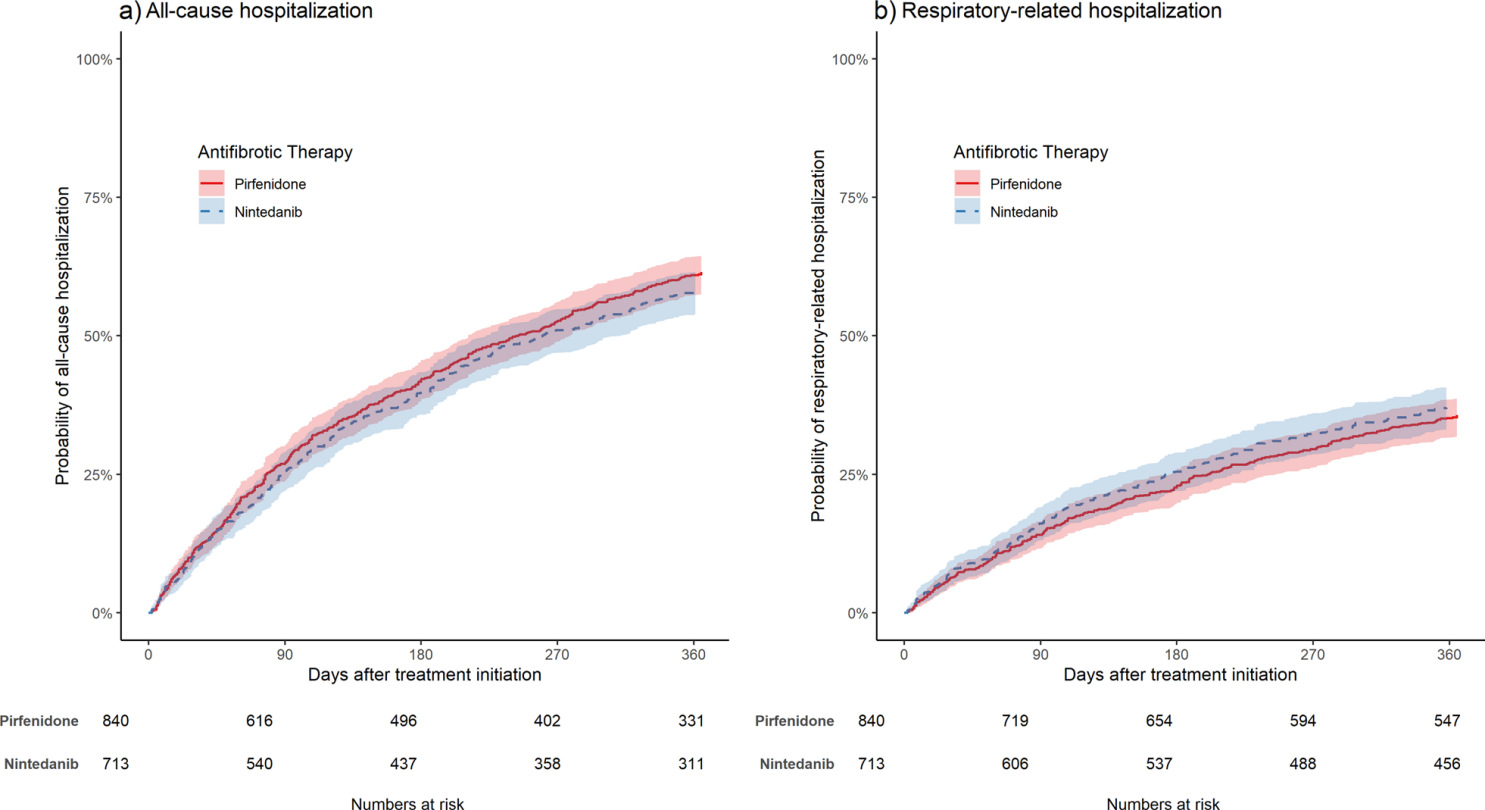
IPTW-weighted cumulative probability curves in patients treated with pirfenidone or nintedanib. **a** comparison of risk for all-cause hospitalization, **b** comparison of risk for respiratory-related hospitalization. The colored areas represent 95% confidence intervals

About 61% of pirfenidone-treated and 57% of nintedanib-treated patients were hospitalized during the first year after treatment initiation. Respiratory-related hospitalizations were experienced by 36% of pirfenidone-treated and 36% of nintedanib-treated patients. Weighted all-cause hospitalization rates were 101.3 (95% CI: 92.8; 110.4) per 100 person-years for pirfenidone-treated and 92.7 (95%-CI: 84.0; 102.0) for nintedanib-treated patients, while respiratory-related hospitalization rates were 45.7 (95% CI: 40.7; 51.2) and 48.6 (95% CI: 42.9; 54.8). There were no substantial differences regarding one-year all-cause as well as respiratory-related hospitalization between both groups (Fig. 3). Accordingly, the weighted HRs for all-cause (HR: 1.09, 95% CI: 0.95; 1.25) and respiratory-related hospitalization (HR: 0.89, 95% CI: 0.72; 1.08) were not significant.

The most common primary diagnoses for all-cause hospitalizations based on the ICD-10 chapters were diseases of the respiratory system [J00-J99], which accounted for 48.2% of hospitalizations of pirfenidone-treated patients and 51.5% of hospitalizations of nintedanib-treated patients. Further key reasons were diseases of the circulatory system [I00-I99] (pirfenidone: 15.8%, nintedanib: 16.6%) and diseases of the digestive system [K00-K93] (pirfenidone: 6.1%, nintedanib: 8.3%). The three most common primary diagnosis for respiratory-related hospitalizations were IPF (pirfenidone: 62.8%, nintedanib: 64.6%), respiratory infection (pirfenidone: 19.8%, nintedanib: 16.3%), and pulmonary hypertension/right heart disease (pirfenidone: 10.7%, nintedanib: 9.7%).

The unweighted results are presented in Additional file 2: Tables S2, S3.

### Costs

We observed no differences in overall (€− 807; 95% CI: €− 2977; €1220) and respiratory-related costs (€− 1282; 95% CI: €− 3423; €534), but at the level of the distinct cost components, outpatient costs were significantly lower for pirfenidone-treated patients (€− 164; 95% CI: €− 280; €− 55) (Table 2). The unweighted results are presented in Additional file 2: Table S4.

**Table 2:** IPTW-weighted one-year costs after treatment initiation in patients treated with pirfenidone or nintedanib and related cost differences with bootstrapped 95% confidence intervals

	Pirfenidone (N = 840)	Nintedanib (N = 713)	
	Costs (in €)	Costs (in €)	Difference (in €)
Overall			
Total	33,893 (32,559; 35,251)	34,700 (33,407; 36,636)	− 807 (− 2977; 1220)
Inpatient	5991 (5247; 7125)	5590 (4739; 7376)	400 (− 1130; 1849)
Outpatient	1107 (1065; 1160)	1271 (1187; 1410)	**− 164 (− 280; − 55)**
Pharmaceuticals	26,796 (25,706; 28,008)	27,839 (26,626; 28,880)	− 1043 (− 2638; 476)
Respiratory-related			
Total	29,085 (27,826; 30,344)	30,366 (29,245; 32,082)	− 1282 (− 3423; 534)
Inpatient	2867 (2369; 3717)	3,096 (2398; 5163)	− 229 (− 1661; 884)
Outpatient	690 (666; 717)	693 (666; 726)	− 3 (− 42; 37)
Pharmaceuticals	25,528 (24,416; 26,587)	26,578 (25,443; 27,710)	− 1050 (− 2549; 473)

Statistically significant results are marked in bold

Bootstrapping with 1000 repetitions, bias-corrected and accelerated bootstrap method

### Sensitivity analysis 1

After excluding pirfenidone-treated patients before 2015, 556 pirfenidone-treated patients were compared to the 713 nintedanib-treated patients. The results of SA1 mirrored the results of the main analysis (See Additional file 3: Tables S5–S7).

### Sensitivity analysis 2

Treatment was discontinued by 50.7% of pirfenidone-treated patients and by 44.0% of nintedanib-treated patients. Among those who discontinued treatment, 14.6% of pirfenidone-treated patients and 5.9% of nintedanib-treated patients switched medications within the treatment gap range of 60 days. Due to the censoring, mortality rates were lower compared to the main analysis (See Additional file 4: Table S8). There were no other noteworthy differences regarding mortality, hospitalization, and costs compared to the main analysis (See Additional file 4: Tables S9, S10).

## Discussion

In Germany, two drugs are currently licensed for the treatment of IPF. However, data on a direct comparison of both drugs in terms of effectiveness and associated costs are sparse. Hence, our study compared pirfenidone and nintedanib-treated IPF patients by using a large claims data set. We found no differences between both drugs regarding two-year all-cause mortality, one-year all-cause and respiratory-related hospitalization, and overall as well as respiratory-related health care costs.

Our finding on similar risk of all-cause mortality within both groups is in line with most published studies comparing pirfenidone and nintedanib-treated patients. Two Network Meta-Analysis reported that pirfenidone and nintedanib-treated patients do not differ in survival [29, 30]. However, these studies did not compare the drugs in one study sample, but merged information from different RCTs to indirectly compare pirfenidone and nintedanib. In an Italian study, a retrospective analysis with 263 IPF patients treated between 2011 and 2019 was conducted, which also showed no differences in survival between the groups [11]. However, the study relied on a small sample and included data only from one specialized Italian center for rare lung diseases. In contrast, our study included information from all over Germany and was not limited to specialized institutions. In another real-world study in the United States with data from 2014 to 2018 comparing 662 pirfenidone with 593 nintedanib-treated patients, no differences in survival were observed between the two drugs [12]. The population included was comparable to ours in terms of space of time, data source (insurance data base), and age of patients. However, a recently published study with data from the French National Health System reported a greater risk of all-cause mortality in nintedanib-treated patients [13]. The study covered a similar time horizon, sample size, population’s age, and sex distribution as our study. Furthermore, the drugs became available at different time points as it was the case in Germany. The study reported a HR of 1.8 (CI 95%: 1.3–2.6) to the disadvantage of nintedanib. Contrary to our analyses, the authors did not perform an intention-to treat analysis, but treatment discontinuation was considered as censoring event. Higher discontinuation rates in pirfenidone-treated patients increased censoring compared to nintedanbib-treated patients, which might to some extent explain the more favorable results in pirfenidone-treated patients. In our SA 2, we also considered treatment discontinuation, which was more frequent in pirfenidone-treated patients. The HR became slightly lower in favor of pirfenidone, but the difference was not significant. Previous evidence already demonstrated the effectiveness of pirfenidone [8, 12, 31] and nintedanib [12, 31] regarding reductions of respiratory-related and all-cause hospitalization. A direct comparison between both drugs in regard of all-cause hospitalization was so far only conducted by Corral et al. [15]. Contrary to our study, these analyses unveiled an advantage of pirfenidone treatment. This might be partially explained by a different study population. Indeed the claims data based study by Corral and colleagues included only patients aged 67 years and older, whereas in our study 24.5% of the patients were younger than 67 years. Also, the proportion of women in their study was higher by approximately 10% compared to our study. Additionally, in their main analysis they considered treatment discontinuation in a similar way as we did in the SA2. Nevertheless, we also found no significant group differences in SA2. One possible explanation is that the study by Corral et al. did not include the hospitalization rate prior to the index date as a covariate in the propensity score model. Although they descriptively reported respiratory-related hospitalization in the three months prior to the index date, they did not report the all-cause hospitalization and did not include the variable in the propensity score model. Also, Corral et al. reported a high rate of administrative censoring due to various reasons, such as discontinuation of the treatment or end of enrollment. In our study, all patients included in the analysis had a post-observation period after treatment initiation of at least one year. Hence, both study designs differ and thus a direct comparison of the results obtained is a sensitive issue. Furthermore, previous evidence suggests an association between hospitalization and mortality in patients with IPF [8, 32, 33]. Therefore, reduced hospitalization rates should be associated with lower mortality rates. However, Corral et al. did not investigate survival differences between drugs and we therefore cannot compare the results in this regard.

Corral et al. also described a lower respiratory-related hospitalization risk for pirfenidone-treated patients with a reported HR of 0.71 (95% CI: 0.57; 0.90). Similarly, Belhassen et al. also reported a greater risk of respiratory-related hospitalizations for nintedanib-treated patient with an HR of 1.3 (95% CI: 1.0; 1.7) [13]. The different results for respiratory-related hospitalizations compared to our study might be explained by different definitions of the outcome, as different conditions were considered to be respiratory-related in each study. Other possible reasons were already mentioned in the discussion above.

Concerning health care expenditures, we did not observe differences between pirfenidone and nintedanib-treated patients in overall and also in respiratory-related costs. Regarding the subcategories, a significant difference in outpatient costs to the benefit of pirfenidone was observed. Nevertheless, since the absolute difference (€164) is rather low when compared to total costs of care, its practical relevance is marginal. Pharmaceutical costs were the main cost drivers for both groups. In our SAs, where we adjusted the time period and excluded those patients who discontinued treatment, also no differences between the groups were observed. The study conducted by Corral et al. [15] was the only other real-world study comparing costs between pirfenidone and nintedanib-treated patients. Their study unveiled significantly lower costs in patients treated with pirfenidone, which was driven by lower inpatient costs, due to lower hospitalization rates. Our study does not support this finding, as we observed no differences in hospitalizations and, consequently, no differences in inpatient costs.

Our sensitivity analyses revealed no substantial differences compared to the main analysis. In SA2 the treatment discontinuation was rather high, with 50.7% of pirfenidone-treated and 44.0% of nintedanib-treated patients. Similar high discontinuation rates were also reported in other claims data based studies [12, 13, 34]. One possible explanation for the difference in the discontinuation rates between the drugs in our study is that nintedanib was approved during the study period. Drug switching was more common in pirfenidone-treated patients with 14.6% compared to 5.9% in nintedanib-treated patients, which could be due to the fact that the new treatment with nintedanib was considered more promising.

When interpreting our results, some limitations need to be considered. First, we used the ICD-10 code J84.1 to identify patients with IPF, which might lead to misidentification. Although pirfenidone was approved only for the treatment of IPF and we used only nintedanib with the tradename “Ofev” to identify relevant patients, some uncertainty regarding the coding remains. Second, we applied the stabilized IPTW approach to adjust for observed confounding, but we could not adjust for important variables such as severity of the disease, socio-economic parameters, and lifestyle factors including smoking history. We assume that the severity of the disease is similar between both groups since there is no guideline recommendation as to which of the drugs should be preferred. When looking into real-world studies, forced vital capacity (FVC) or oxygen use were mostly balanced between pirfenidone and nintedanib-treated patients [12, 15, 34, 35]. As a rare example in the opposite direction, differences were reported in one Italian study showing worse FVC baseline values for nintedanib [11]. Against this background, group differences regarding concomitant oxygen therapy or FVC values in our sample cannot be fully excluded. Third, we could not distinguish between IPF-related and all-cause mortality. When comparing the drugs, it would have been important to investigate IPF-related mortality.

There are also several strength of our study. Claims data are less prone to selection bias compared to primary data, which in the context of IPF often is collected in registers and may include predominantly patients treated in specialized hospitals. IPF is a very rare disease and data sources like ours are needed to achieve a sufficiently large population. Furthermore, this is one of the very few studies comparing pirfenidone and nintedanib directly in a large, widely unselected IPF population.

## Conclusion

Pirfenidone and nintedanib appear to be associated with equivalent mortality, hospitalization, and SHI-borne costs. These effect parameters however disregard patient-relevant outcomes such as quality of life and clinically relevant aspects such as lung function and adverse effects. Therefore, the decision to treat with pirfenidone and nintedanib should be made on a case-by-case basis taking into account clinical characteristics including comorbidities and comedication, individual risk for adverse events, and patient preferences.

## Supplementary Information

**Additional file 1: Table S1.** Drug-related ATC-Codes for coding of respiratory-related outcomes and covariables.

**Additional file 2: Table S2.** Unweighted incidence rates for mortality and hospitalization of the main analysis. **Table S3.** Unweighted Cox Proportional Hazard models for two-year mortality and one-year hospitalization of the main analysis. **Table S4.** Unweighted one-year costs and cost differences with bootstrapped 95% confidence intervals of the main analysis.

**Additional file 3: Table S5.** Sensitivity analysis 1 with unweighted and IPTW-weighted incidence rates for mortality and hospitalization. **Table S6.** Sensitivity analysis 1 with unweighted and IPTW-weighted Cox Proportional Hazard models for 2 year mortality and 1-year hospitalization. **Table S7.** Sensitivity analysis 1 with unweighted and IPTW-weighted cost differences with bootstrapped 95% confidence intervals.

**Additional file 4: Table S8.** Sensitivity analysis 2 with unweighted and IPTW-weighted incidence rates for mortality and hospitalization. **Table S9.** Sensitivity analysis 2 with unweighted and IPTW-weighted Cox Proportional Hazard models for 2 year mortality and 1-year hospitalization. **Table S10.** Sensitivity analysis 2 with unweighted and IPTW-weighted cost differences with bootstrapped 95% confidence intervals.

## Data Availability

The authors confirm that the data utilized in this study cannot be made available in the manuscript, the supplemental files, or in a public repository due to German data protection laws (‘Bundesdatenschutzgesetz’, BDSG). Therefore, they are stored on a secure drive in the AOK Research Institute (WIdO) to facilitate replication of the results. Generally, access to data of statutory health insurance funds for research purposes is possible only under the conditions defined in German Social Law (SGB V § 287). Requests for data access can be sent as a formal proposal specifying the recipient and purpose of the data transfer to the appropriate data protection agency. Access to the data used in this study can only be provided to external parties under the conditions of the cooperation contract of this research project and after written approval by the health insurance. For assistance in obtaining access to the data, please contact wido@wido.bv.aok.de.

## Abbreviations

AOK: Allgemeine Ortskrankenkasse
CI: Confidence interval
FVC: Forced vital capacity
GIMD: German Index of Multiple Deprivation
HR: Hazard ratio
ILD: Interstitial lung disease
IPF: Idiopathic pulmonary fibrosis
IPTW: Inverse probability of treatment weighting
RCT: Randomized controlled trials
SA: Sensitivity analysis
SHI: Statutory Health Insurance
SMD: Standardized Mean Difference
