# Supplementary material for: Pirfenidone vs. nintedanib in patients with idiopathic pulmonary fibrosis: a retrospective cohort study
Source: Respir Res. 2021 Oct 19;22:268. doi: 10.1186/s12931-021-01857-y (PMC8527681; doi:10.1186/s12931-021-01857-y)
Supplement: Supplementary file 1 — Additional file 1: Table S1. Drug-related ATC-Codes for coding of respiratory-related outcomes and covariables. [file 12931_2021_1857_MOESM1_ESM.docx]

Additional file 1: Table S1 Drug-related ATC-Codes for coding of respiratory-related outcomes and covariables

| **Drugs** | **ATC-Codes** |
| --- | --- |
| Pirfenidone | L04AX05 |
| Nintedanib | L01XE31 |
| Immunosuppressants | L01XC02, L01AA01, L04AA06, L04AA10, L04A13, L04AB01, L04AB02, L04AB03, L04AB04, L04AB05, L04AB06, L04AC01, L04AC02, L04AC03, L04AC04, L04AC05, L04AC07, L04AC08, L04AC09, L04AC10, L04AC11, L04AC12, L04AX01, L04AX03 |
| Acetylcysteine | R05CB01 |
| Glucocorticoids, Corticosteroids | H02AB, R01AD |
| Sildenafil | G04BE03 |
| Antihypertensives for pulmonary arterial hypertension | C02KX |
| Treatment of cardiovascular disease | C10AA, C07, C09AA, C09B, C09CA, C09D |
| Treatment with anti-clotting drug | B01AC, B01AB, B01AA |
| Treatment with anti-acid drugs | A02A, A02BC, A02BA |
| Treatment with anti-depressants | N06A |
| Treatment with anti-diabetic drugs | A10A, A10B |
| Treatment with drugs against obstructive airway disease | R03AC03, R03AC12, R03AC13, R03AC18, R03AC19, R03BB01, R03BB02, R03BB03, R03BB04, R03BB05, R03BB06, R03BB07, R03BA01, R03BA02, R03BA03, R03BA04, R03BA05, R03BA06, R03BA07, R03BA08, R03BA09, R03AK06, R03AK07, R03AK08, R03AK10, R03AK11, R03AL03, R03AL04, R04AL05, R03AL06 |
| Treatment of heart insufficiency/cardiac arrhythmia | C01AA, C01BD, C03 |
