# Supplementary material for: Pirfenidone vs. nintedanib in patients with idiopathic pulmonary fibrosis: a retrospective cohort study
Source: Respir Res. 2021 Oct 19;22:268. doi: 10.1186/s12931-021-01857-y (PMC8527681; doi:10.1186/s12931-021-01857-y)
Supplement: Supplementary file 2 — Additional file 2: Table S2. Unweighted incidence rates for mortality and hospitalization of the main analysis. Table S3. Unweighted Cox Proportional Hazard models for two-year mortality and one-year hospitalization of the main analysis. Table S4. Unweighted one-year costs and cost differences with bootstrapped 95% confidence intervals of the main analysis. [file 12931_2021_1857_MOESM2_ESM.docx]

Additional file 2: Table S2 Unweighted incidence rates for mortality and hospitalization of the main analysis

|  | *Unweighted* | |
| --- | --- | --- |
|  | *IR* | *(95%-CI)* |
| **Incidence rate per 100 person-years** |  |  |
| *All-cause mortality* |  |  |
| Pirfenidone-treated patients (N = 840) | 21.9 | (19.5; 24.5) |
| Nintedanib-treated patients (N = 713) | 25.0 | (22.1; 28.1) |
| *All-cause hospitalization* |  |  |
| Pirfenidone-treated patients (N = 840) | 99.9 | (91.5; 108.9) |
| Nintedanib-treated patients (N = 713) | 88.7 | (80.3; 97.8) |
| *Respiratory-related hospitalization* |  |  |
| Pirfenidone-treated patients (N = 840) | 45.3 | (40.3; 50.7) |
| Nintedanib-treated patients (N = 713) | 46.6 | (41.1; 52.7) |

*CI: confidence interval, IR: Incidence rate*

Additional file 2: Table S3 Unweighted Cox Proportional Hazard models for two-year mortality and one-year hospitalization of the main analysis

|  | *Unweighted* | |
| --- | --- | --- |
|  | *HR* | *(95%-CI)* |
| *2-year all-cause mortality* |  |  |
| Pirfenidone (N = 840) vs. Nintedanib (N = 713) | 0.88 | (0.76; 1.07) |
| *1-year all-cause hospitalization* |  |  |
| Pirfenidone (N = 840) vs. Nintedanib (N = 713) | 1.12 | (0.98; 1.27) |
| *1-year respiratory-related hospitalization* |  |  |
| Pirfenidone (N = 840) vs. Nintedanib (N = 713) | 0.97 | (0.82; 1.14) |

*CI: confidence interval, HR: Hazard ratio*

Additional file 2: Table S3 Unweighted one-year costs and cost differences with bootstrapped 95% confidence intervals of the main analysis

|  | Pirfenidone (N = 840) | Nintedanib (N = 713) |  |
| --- | --- | --- | --- |
|  | Costs (in €) | Costs (in €) | *Difference (in €)* |
| **Overall** |  |  |  |
| Total | 34,230 (32,849; 35,658) | 34,351 (32,989; 36,096) | -121 (-2,179; 1,849) |
| Inpatient | 6,176 (5,278; 7,279) | 5,506 (4,481; 6,910) | 670 (-918; 2,179) |
| Outpatient | 1,127 (1,083; 1,176) | 1,264 (1,175; 1,380) | **-137 (-248; -28)** |
| Pharmaceuticals | 26,927 (25,836; 27,921) | 27,582 (26,587; 28,607) | -655 (-2,155; 738) |
|  |  |  |  |
| **Respiratory-related** |  |  |  |
| Total | 29,316 (28,113; 30,543) | 30,114 (28,773; 31,733) | -798 (-2,767; 1,167) |
| Inpatient | 2,981 (2,315; 3,828) | 3,096 (2,235; 4,444) | -114 (-1,678; 1,243) |
| Outpatient | 691 (668; 717) | 697 (670; 726) | -6 (-46; 34) |
| Pharmaceuticals | 25,644 (24,592; 26,706) | 26,321 (25,394; 27,358) | -678 (-2,171; 763) |

*Bootstrapping with 1000 repetitions, bias-corrected and accelerated bootstrap method*
