# Supplementary material for: Pirfenidone vs. nintedanib in patients with idiopathic pulmonary fibrosis: a retrospective cohort study
Source: Respir Res. 2021 Oct 19;22:268. doi: 10.1186/s12931-021-01857-y (PMC8527681; doi:10.1186/s12931-021-01857-y)
Supplement: Supplementary file 3 — Additional file 3: Table S5. Sensitivity analysis 1 with unweighted and IPTW-weighted incidence rates for mortality and hospitalization. Table S6. Sensitivity analysis 1 with unweighted and IPTW-weighted Cox Proportional Hazard models for 2 year mortality and 1-year hospitalization. Table S7. Sensitivity analysis 1 with unweighted and IPTW-weighted cost differences with bootstrapped 95% confidence intervals. [file 12931_2021_1857_MOESM3_ESM.docx]

Additional file 3: Table S5 Sensitivity analysis 1 with unweighted and IPTW-weighted incidence rates for mortality and hospitalization

|  | *Unweighted* | | *Weighted* | |
| --- | --- | --- | --- | --- |
|  | *IR* | *(95%-CI)* | *IR* | *(95%-CI)* |
| **Incidence rate per 100 person-years** |  |  |  |  |
| *All-cause mortality* |  |  |  |  |
| Pirfenidone-treated patients (N = 840) | 21.6 | (18.7; 24.9) | 21.1 | (18.2; 24.3) |
| Nintedanib-treated patients (N = 713) | 25.0 | (22.1; 28.1) | 25.0 | (22.2; 28.2) |
| *All-cause hospitalization* |  |  |  |  |
| Pirfenidone-treated patients (N = 840) | 101.3 | (91.5; 108.9) | 102.0 | (91.5; 113.4) |
| Nintedanib-treated patients (N = 713) | 88.7 | (80.3; 97.8) | 92.0 | (83.3; 101.3) |
| *Respiratory-related hospitalization* |  |  |  |  |
| Pirfenidone-treated patients (N = 840) | 45.7 | (39.5; 52.5) | 45.3 | (39.2; 52.1) |
| Nintedanib-treated patients (N = 713) | 46.6 | (41.1; 52.7) | 48.0 | (42.4; 54.2) |

*CI: confidence interval, IR: Incidence rate*

Additional file 3: Table S6 Sensitivity analysis 1 with unweighted and IPTW-weighted Cox Proportional Hazard models for 2-year mortality and 1-year hospitalization

|  | *Unweighted* | | *Weighted* | |
| --- | --- | --- | --- | --- |
|  | *HR* | *(95%-CI)* | *HR* | *(95%-CI)* |
| *2-year all-cause mortality* |  |  |  |  |
| Pirfenidone (N = 840) vs. Nintedanib (N = 713) | 0.87 | (0.72; 1.04) | 0.85 | (0.70; 1.02) |
| *1-year all-cause hospitalization* |  |  |  |  |
| Pirfenidone (N = 840) vs. Nintedanib (N = 713) | 1.13 | (0.98; 1.31) | 1.10 | (0.95; 1.28) |
| *1-year respiratory-related hospitalization* |  |  |  |  |
| Pirfenidone (N = 840) vs. Nintedanib (N = 713) | 0.98 | (0.81; 1.18) | 0.94 | (0.78; 1.14) |
| *CI: confidence interval, H­­­­R: Hazard Ratio* | | | | |

Additional file 3: Table S7 Sensitivity analysis 1 with unweighted and IPTW-weighted cost differences with bootstrapped 95% confidence intervals

| **Sensitivity analysis 1: Patients included after approval of both drugs (year 2015 and later)** | | |
| --- | --- | --- |
|  | Unweighted | Weighted |
|  | *Difference (in €)* | *Difference (in €)* |
| **Overall** |  |  |
| Total | 149 (-2,105; 2,242) | -338 (-2,427; 1,750) |
| Inpatient | 301 (-1,644; 1,817) | 66 (-1,397; 1,662) |
| Outpatient | **-122 (-243; -13)** | **-152 (-274; -33)** |
| Pharmaceuticals | -30 (-1,606; 1,654) | -251 (-1,849; 1,381) |
|  |  |  |
| **Respiratory-related** |  |  |
| Total | -172 (-2,204; 1,683) | -460 (-2,465; 1,498) |
| Inpatient | -47 (-1,382; 1,301) | -160 (-1,610; 948) |
| Outpatient | -4 (-40; 35) | -6 (-46; 32) |
| Pharmaceuticals | -120 (-1,717; 1,435) | -294 (-2,086; 1,358) |

*Bootstrapping with 1000 repetitions, bias-corrected and accelerated bootstrap method*
