# Supplementary material for: Pirfenidone vs. nintedanib in patients with idiopathic pulmonary fibrosis: a retrospective cohort study
Source: Respir Res. 2021 Oct 19;22:268. doi: 10.1186/s12931-021-01857-y (PMC8527681; doi:10.1186/s12931-021-01857-y)
Supplement: Supplementary file 4 — Additional file 4: Table S8. Sensitivity analysis 2 with unweighted and IPTW-weighted incidence rates for mortality and hospitalization. Table S9. Sensitivity analysis 2 with unweighted and IPTW-weighted Cox Proportional Hazard models for 2 year mortality and 1-year hospitalization. Table S10. Sensitivity analysis 2 with unweighted and IPTW-weighted cost differences with bootstrapped 95% confidence intervals. [file 12931_2021_1857_MOESM4_ESM.docx]

Additional file 4: Table S8 Sensitivity analysis 2 with unweighted and IPTW-weighted incidence rates for mortality and hospitalization

|  |  | |  | |
| --- | --- | --- | --- | --- |
|  | *Unweighted* | | *Weighted* | |
|  | *IR* | *(95%-CI)* | *IR* | *(95%-CI)* |
| **Incidence rate per 100 person-years** |  |  |  |  |
| *All-cause mortality* |  |  |  |  |
| Pirfenidone-treated patients (N = 840) | 19.2 | (16.2; 22.7) | 19.3 | (16.2; 22.7) |
| Nintedanib-treated patients (N = 713) | 22.7 | (19.1; 26.7) | 22.6 | (19.1; 26.7) |
| *All-cause hospitalization* |  |  |  |  |
| Pirfenidone-treated patients (N = 840) | 107.5 | (97.2; 118.5) | 109.4 | (99.0; 120.6) |
| Nintedanib-treated patients (N = 713) | 90.1 | (80.4; 100.7) | 93.8 | (83.9; 104.6) |
| *Respiratory-related hospitalization* |  |  |  |  |
| Pirfenidone-treated patients (N = 840) | 46.5 | (40.7; 52.9) | 45.3 | (39.2; 52.2) |
| Nintedanib-treated patients (N = 713) | 46.7 | (40.9; 53.1) | 47.7 | (41.4; 54.7) |

*CI: confidence interval, IR: Incidence rate*

Additional file 4: Table S9 Sensitivity analysis 2 with unweighted and IPTW-weighted Cox Proportional Hazard models for 2-year mortality and 1-year hospitalization

|  | *Unweighted* | | *Weighted* | |
| --- | --- | --- | --- | --- |
|  | *HR* | *(95%-CI)* | *HR* | *(95%-CI)* |
| *2-year all-cause mortality* |  |  |  |  |
| Pirfenidone (N = 840) vs. Nintedanib (N = 713) | 0.85 | (0.67; 1.07) | 0.85 | (0.67; 1.09) |
| *1-year all-cause hospitalization* |  |  |  |  |
| Pirfenidone (N = 840) vs. Nintedanib (N = 713) | **1.17** | **(1.01; 1.36)** | 1.14 | (0.98; 1.34) |
| *1-year respiratory-related hospitalization* |  |  |  |  |
| Pirfenidone (N = 840) vs. Nintedanib (N = 713) | 1.02 | (0.84; 1.23) | 0.97 | (0.79; 1.19) |
| *CI: confidence interval, H­­­­R: Hazard Ratio* | | | | |

Additional file 4: Table S10 Sensitivity analysis 2 with unweighted and IPTW-weighted cost differences with bootstrapped 95% confidence intervals

| **Sensitivity analysis 2: Discontinuation of drugs (treatment gap > 60 days) – costs per month** | | |
| --- | --- | --- |
|  | Unweighted | Weighted |
|  | *Difference (in €)* | *Difference (in €)* |
| **Overall** |  |  |
| Total | **-233 (-430; -70)** | -136 (-325; 47) |
| Inpatient | -78 (-221; 31) | -30 (-173; 85) |
| Outpatient | **-21 (-64; 16)** | -4 (-59; 38) |
| Pharmaceuticals | **-135 (-250; -4)** | -102 (-226; 20) |
|  |  |  |
| **Respiratory-related** |  |  |
| Total | **-220 (-403; -83)** | -139 (-312; 12) |
| Inpatient | -66 (-187; 21) | -40 (-163; 50) |
| Outpatient | -3 (-18; 14) | 14 (-5; 32) |
| Pharmaceuticals | **-151 (-266; -44)** | -112 (-225; 11) |

*Bootstrapping with 1000 repetitions, bias-corrected and accelerated bootstrap method*
